# Research on the correlation between the renal resistive index, renal microvessel density, and fibrosis

**DOI:** 10.1080/0886022X.2023.2273423

**Published:** 2023-10-24

**Authors:** Jingping Wu, Jian Liu, Guanghan Li, Weiliang Sun, Jiang Liu, Wenge Li, Hao Wang, Min Zheng

**Affiliations:** a Department of Ultrasound Medicine, China-Japan Friendship Hospital, Beijing, China; b Institute of Clinical Medical Sciences, China-Japan Friendship Hospital, Beijing, China; c China-Japan Friendship School of Clinical Medicine, Peking University, Beijing, China; d Department of Nephrology, China-Japan Friendship Hospital, Beijing, China; e Department of Radiation Oncology, China-Japan Friendship Hospital, Beijing, China

**Keywords:** Ultrasonography, chronic kidney disease, fibrosis, microvessel density, renal resistive index

## Abstract

**Background:**

This study was designed to investigate the relationship between the renal resistive index (RRI), renal microvessel density (RMD), and fibrosis in patients with chronic kidney disease (CKD).

**Methods:**

A total of 73 CKD patients were included in the study. Prior to kidney biopsy, we recorded the RRI of the interlobar artery and the estimated glomerular filtration rate (eGFR). Immunohistochemical analysis was performed to assess CD34 expression, and Masson staining was used to evaluate histopathological specimens for RMD and the degree of fibrosis. The percentage of the positive area (PPA) was recorded. Subsequently, we investigated the correlation between RRI, RMD, and kidney fibrosis.

**Results:**

RMD (CD34 PPA-total and CD34 PPA-peritubular capillary) showed a slight increase in early CKD stages (1-2) and gradually declined from CKD stages 2 to 5. No correlation was observed between the RRI and RMD or between the RRI and fibrosis across CKD stages 1 to 5. However, across CKD stages 2 to 5, RRI negatively correlated with CD34 PPA-glomerulus (r = −0.353, *p* = 0.022), but no correlation was found with CD34 PPA-total, CD34 PPA-peritubular capillary, or kidney fibrosis. eGFR showed a positive correlation with RMD (CD34 PPA-total, CD34 PPA-peritubular capillary, and CD34 PPA-glomerulus) across CKD stages 2 to 5, while no correlation was found from CKD stages 1 to 5.

**Conclusion:**

There was no correlation between RRI and RMD or between RRI and fibrosis across CKD stages 1 to 5 (RRI ≤ 0.7).

## Introduction

Chronic kidney disease (CKD) is characterized by progressive and irreversible damage to kidney structure and function, and it has a multifactorial etiology. A study conducted in 2016 reported the global mean prevalence of all CKD stages as 13.4% (ranging from 11.7% to 15.1%), with stages 3–5 accounting for 10.6% (ranging from 9.2% to 12.2%) [1]. As a result, there is considerable research interest in the early detection of kidney diseases. The renal resistive index (RRI) is mostly used as a prognostic tool in CKD. Various extrarenal factors, such as systemic pulse pressure [2], aortic stiffness [3], and heart rate [4], have been found to influence RRI. Conversely, studies have also established a strong correlation between RRI and the degree of kidney disease, suggesting its potential as a predictor of kidney disease progression [5–13]. A long-term follow-up study demonstrated that only 2.0% of patients with a normal initial estimated glomerular filtration rate (eGFR) and RRI (eGFR ≥60 mL/min, RRI <0.70) experienced kidney failure progression, compared to 65.0% of patients with an abnormal initial eGFR and RRI (eGFR <60 mL/min, RRI ≥0.70) [13].

CKD progression is characterized by an increase in kidney tissue fibrosis and a reduction in blood supply [14,15]. Therefore, it is crucial to determine whether there is a correlation between the renal resistive index (RRI) and both fibrosis and kidney blood supply. While some authors have observed a correlation between RRI and fibrosis [16,17], a correlation between RRI and peritubular capillary density has been reported from only one small-scale study (*n* = 30) [17]. No existing study has reported data on the correlation between RRI and glomerular vascular density. Consequently, we conducted the present study based on these assumptions. We utilized CD34 to label renal vascular endothelial cells (ECs) and measured the renal microvessel density (RMD) of the glomerulus, peritubular capillary, and total RMD (glomerulus and peritubular capillary) in patients across various stages of CKD. Additionally, we used Masson staining to quantify the degree of kidney fibrosis. Subsequently, we investigated the correlation between RRI and RMD as well as between RRI and the degree of kidney fibrosis.

## Materials and methods

### Study population

This prospective study was conducted between August 2019 and January 2020 and received approval from the Ethics Committee of the China-Japan Friendship Hospital (2019-52-K33). The study adhered to the principles outlined in the Helsinki Declaration of 1975, as revised in 2013. A total of 104 patients with suspected kidney disease were included in this study. The inclusion criteria were as follows: (a) meeting the diagnostic criteria for CKD, which included a disease course of more than 3 months and histopathologically confirmed kidney disease [18]; and (b) providing consent for study participation (written informed consent was obtained). The exclusion criteria consisted of the following: (A) diagnosis of acute kidney failure, solitary kidney, sponge kidney, renal vein thrombosis, nutcracker syndrome, renal artery stenosis, kidney tumors or cysts (diameter >3 cm), polycystic kidney, hydronephrosis, and kidney infections; (B) refusal to undergo kidney biopsy; (C) unqualified histological staining that could not be analyzed; (D) inability to hold breath during ultrasonography, thereby affecting the quality of obtained images; (E) unsatisfactory ultrasound images due to intestinal gas, making it impossible to exclude obstructive lesions of the renal artery and/or vein and abdominal aorta; and (F) a history of atrial fibrillation.

A total of 31 patients were excluded from the study: 5 patients lacked satisfactory histopathological specimens for CD34 evaluation, 10 patients did not meet the diagnostic criteria for CKD, 8 patients failed accurate determination of renovascular and abdominal aortic stenosis due to abdominal flatulence, 2 patients did not cooperate and we could not obtain satisfactory RRI data, and 6 patients did not undergo kidney puncture during hospitalization. Ultimately, a total of 73 patients were included in the study.

### CKD classification

The CKD classification was based on The Kidney Disease: Improving Global Outcomes criteria, and the estimated glomerular filtration rate (eGFR) was categorized into five stages as follows: G1: eGFR >90 mL/min, G2: eGFR = 60–89 mL/min, G3: eGFR 30–59 mL/min, G4: eGFR 15–29 mL/min, and G5: eGFR <15 mL/min [18].

### Ultrasonography evaluation

Ultrasonography was conducted prior to kidney biopsy by two experienced operators (Liu J. and Li G.), both of whom had over 5 years of experience in kidney ultrasonography. A Resona 8 ultrasound system (Mindray Bio-Medical Electronics Co., Ltd.) equipped with a convex SC5-1 U transducer was used for the examinations.

Initially, grayscale ultrasound imaging was utilized to assess the size, shape, echotexture, and structure of both kidneys. Subsequently, color Doppler ultrasonography was employed to rule out obstructive diseases in the renal veins, arteries, and abdominal aorta. Furthermore, color Doppler ultrasonography was used to evaluate the main trunk and branches of the renal arteries. During this stage, relevant blood flow parameters such as peak systolic velocity (PSV), end diastolic velocity (EDV), and the renal resistive index (RRI) were recorded. The RRI was calculated using the Pourcelot formula: (PSV - EDV) divided by PSV.

Satisfactory ultrasonic quality control was determined based on the following criteria: clear grayscale ultrasound image, demonstration of intrarenal blood flow distribution, and obtaining at least three Doppler time-velocity spectra for each kidney. The sample gate, pulse repetition frequency, and gain were adjusted to optimize the quality of the ultrasonographic images and measurements [13].

**Figure 1.**
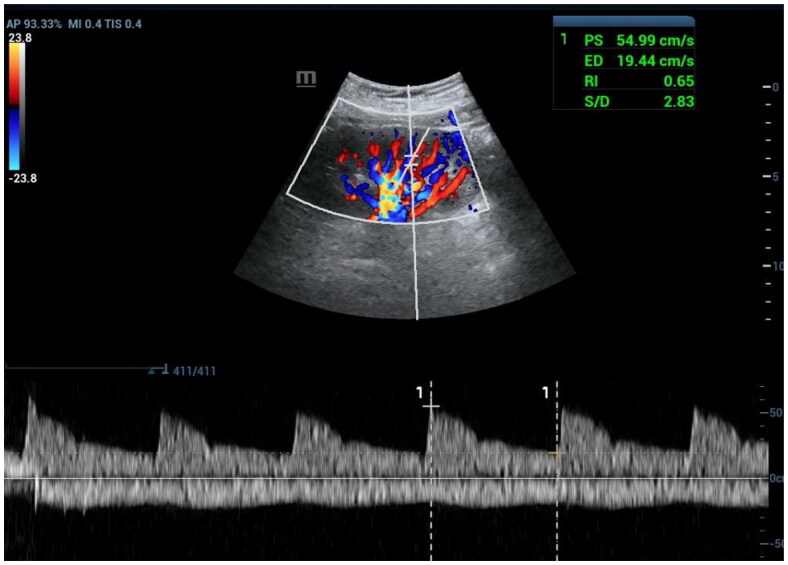
Measurement of RRI. ED: End-diastolic velocity, PS: Peak systolic velocity, RI: Resistive index

The Doppler signal of the interlobar artery was acquired along the border of the medullary pyramid in each kidney (refer to Figure 1). The RRI was measured in three different parts of the same kidney, and the median value was calculated for subsequent statistical analysis.

Before the ultrasonography procedure, relevant patient data, including age, sex, height, weight, systolic blood pressure (SBP), diastolic blood pressure (DBP), and other pertinent information, were recorded.

### Histopathological evaluation

Kidney biopsy samples were obtained from the lower pole of the left kidney parenchyma, with two complete samples collected for each patient. The biopsied kidney tissue was fixed in 10.0% formalin for a minimum of 24 h. After embedding in paraffin, the tissue was cut into 4 μm slices and thoroughly dried. Immunohistochemical staining was conducted as follows: The sections were deparaffinized and rehydrated, and endogenous peroxidase activity was blocked using 0.3% hydrogen peroxide for 15 min. Subsequently, the sections were washed thrice with phosphate-buffered saline (PBS) for 3 min each and incubated overnight with primary CD34 antibody (ab81289, Abcam, plc). After rinsing with PBS thrice (3 min each), the sections were incubated with anti-rabbit immunoglobulin (Ig) G horseradish peroxidase-linked for 2 h (K5007, Dako). Following another round of PBS washes, the sections were incubated with 3,3′-diaminobenzidine for 3 min. Microscopic evaluation was performed after the sections were dehydrated.

For determination of tissue fibrosis, Masson’s trichrome stain was applied according to the standard protocol.

**Figure 2.**
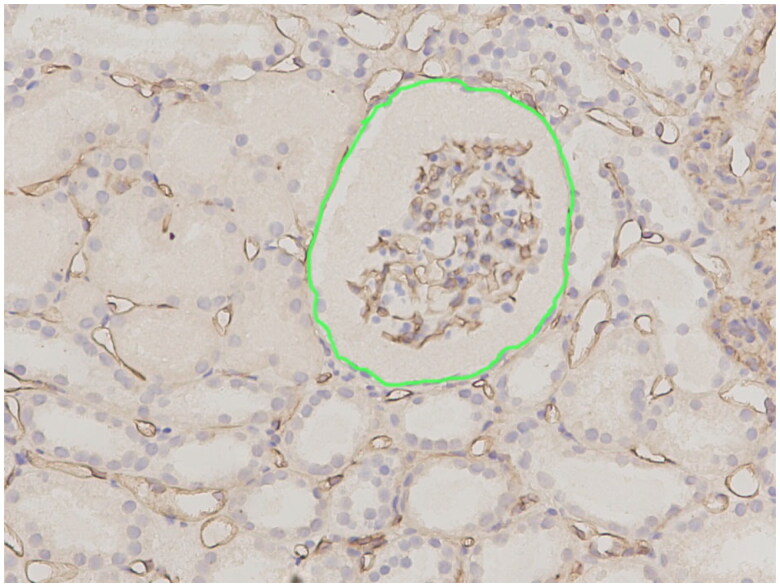
Histopathological findings in CD34-stained specimens showing the distribution of vascular endothelial cells in kidney tissue. Areas of CD34 positivity appear brown. The glomerulus was visualized within the green curve, and the outer area represents the peritubular area. In this view (200×), the percentages of CD34 -stained positive areas of the total area, glomerulus, and peritubular capillary were 8.1%, 12.9%, 7.3%, respectively.

**Figure 3.**
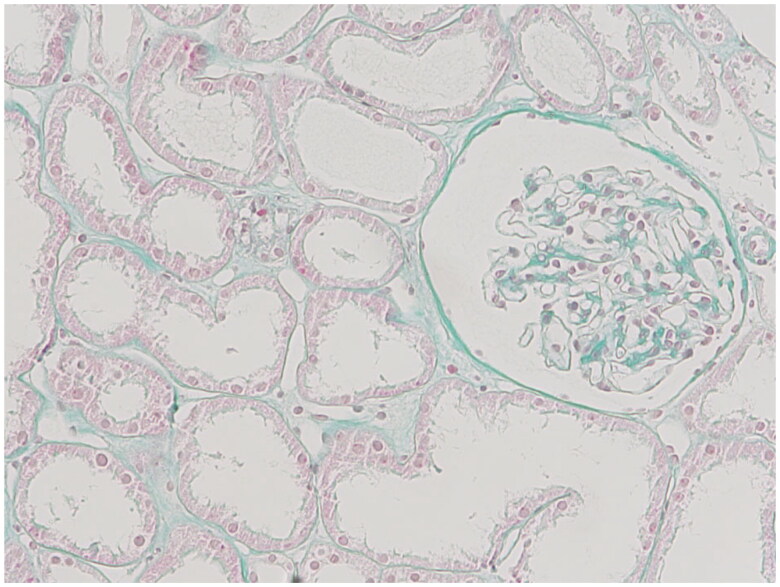
Photomicrograph showing areas stained using the Masson stain. Fibrotic areas appear green. In this view, approximately 10.5% of the specimen represents the fibrotic area.

The renal microvessel density (RMD) was assessed as follows: Images were captured using an optical microscope (BX53, Olympus). Six random fields in the cortical view were selected (original magnification, ×200) for each section, with each field containing at least one glomerulus and no artery. Using Image-Pro Plus software, version 6.0 (Media Cybernetics Inc.), the total area and internal positive area of a visual field were calculated separately. The glomerular boundary was manually delineated, and the total glomerular area and its internal positive area were calculated (refer to Figure 2). Furthermore, the peritubular capillary area and its internal positive area of a visual field were calculated (total area = glomerular area + interstitial area). The sum of the internal positive area divided by the sum of the area (total, peritubular capillary, and glomerulus) and the percentage of positive area (PPA) were calculated for the six fields. The degree of fibrosis for the total area was determined as described earlier (refer to Figure 3). Histopathological evaluation was performed by a pathologist with 10 years of experience (Sun W) who was blinded to the patients’ clinical and ultrasonographic data.

### Clinical data

Data for eGFR was obtained from the hospital information system, and the calculation was according to the Chronic Kidney Disease-Epidemiology Collaboration (CKD-EPI) equation (serum creatinine,SCr).eGFR =144 × (SCr/0.70)^−0.329^ × 0.9929^Age^ (Female,SCr ≤ 0.70^);^ eGFR =144 × (SCr/0.70)^−1.209^ × 0.9929^Age^ (Female,SCr >0.70^);^ eGFR =141 × (SCr/0.90)^−0.411^ × 0.9929^Age^ (Male,SCr ≤ 0.90);eGFR =141 × (SCr/0.90)^−1.209^ × 0.9929^Age^ (Male,SCr > 0.90).

### Statistical analysis

The statistical analysis was conducted using SPSS software, version 21.0 for Windows (IBM Inc.). Measurement data such as SBP, DBP, and kidney length are represented by the mean plus or minus standard deviation. The independent samples t test was utilized for intergroup comparisons of mean values, while Spearman correlation analysis was employed to assess the correlation between the variables. A p value less than 0.05 was considered statistically significant.

## Results

### Patient characteristics

**Figure 4.**
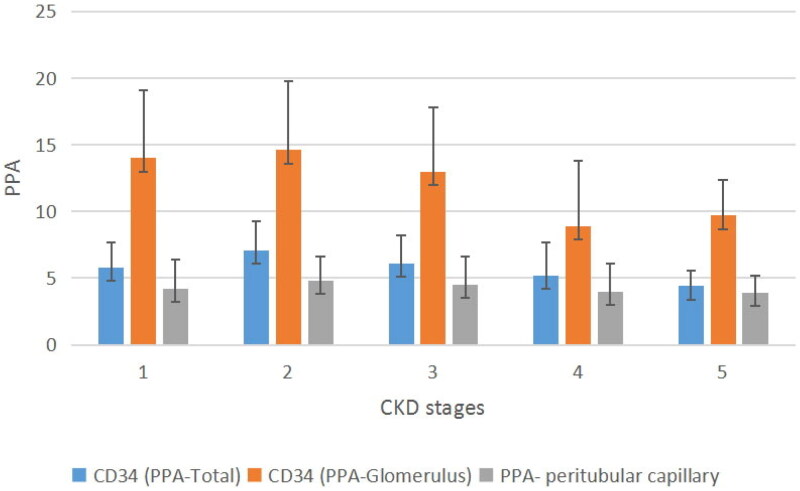
Percentage of the PPA across different CKD stages. CKD: chronic kidney disease; PPA: percentage of the positive area.

Table 1 provides an overview of the histopathological types of CKD. IgA nephropathy and membranous nephropathy accounted for over 50% of the diagnosed cases (53.4%). Age, SBP, DBP, kidney length, body mass index, eGFR, RI of the interlobular artery, Masson stain-documented PPA, CD34 PPA-total, CD34 PPA-glomerulus, and CD34 PPA-peritubular capillary across different stages of CKD are presented in Table 2 and Figure 4.

**Table 1.** Study population data.

Etiology of chronic kidney disease	Mumber (73)
IgA nephropathy	20
Membranous nephropathy	19
Focal segmental glomerulosclerosis	4
Focal proliferative glomerulonephritis	1
Ischemic renal injury	2
Mesangial Proliferative glomerulonephritis	1
Minimal change nephropathy	3
Chronic interstitial nephritis	2
Lupus nephritis	5
Malignant arteriolar nephrosclerosis	1
Diabetic nephropathy	7
Amyloid nephropathy	1
Purpura nephritis	1
Membranous nephropathy & IgA nephropathy	2
Membranous nephropathy & Malignant arteriolar nephrosclerosis	1
IgA nephropathy & Minimal change nephropathy	1
IgA nephropathy & Malignant arteriolar nephrosclerosis	2

**Table 2.** Study data of the 73 patients.

Characteristic	CKD1-5	CKD1	CKD2	CKD3	CKD4	CKD5
**Basic data**						
Number of patients	73	31	19	14	6	3
Males/females	50/23	12/19	4/15	9/5	5/1	2/1
Age (years)	42.9 ± 13.4	40.7 ± 11.8	46.2 ± 13.9	47.7 ± 15.0	39.5 ± 12.3	27.3 ± 3.2
BMI (kg/cm2)	25.6 ± 4.5	24.7 ± 4.5	26.6 ± 4.6	26.3 ± 4.7	25.8 ± 4.1	24.3 ± 6.7
SBP(mmHg)	135.3 ± 17.3	131.3 ± 16.4	138.5 ± 15.1	134.7 ± 20.3	141.7 ± 17.0	145.3 ± 25.7
DBP(mmHg)	90.5 ± 14.5	86.3 ± 10.3	90.9 ± 10.7	95.6 ± 20.6	93.7 ± 22.9	100.7 ± 12.9
Left kidney length(cm)	10.8 ± 0.9	10.9 ± 0.7	10.8 ± 0.8	10.7 ± 1.1	10.9 ± 1.0	9.4 ± 0.7
eGFR (ml/min/1.73m^2^)	75.6 ± 33.6	107.2 ± 11.9	73.8 ± 9.8	44.2 ± 8.5	23.6 ± 3.6	9.7 ± 2.4
Interlobular artery RRI of LK	0.52 ± 0.07	0.52 ± 0.06	0.53 ± 0.06	0.52 ± 0.08	0.53 ± 0.12	0.49 ± 0.07
**Pathological data(LK)**						
Masson (PPA-total)	18.3 ± 7.5%	16.1 ± 6.1%	18.5 ± 8.9%	20.7 ± 5.9%	19.4 ± 7.1%	26.6 ± 12.4%
Masson (PPA-glomerulus)	19.3 ± 7.2%	17.2 ± 7.4%	20.4 ± 14.1%	21.3 ± 6.0%	18.0 ± 6.5%	26.7 ± 15.7%
Masson (PPA- peritubular capillary)	9.7 ± 3.5%	6.0 ± 2.6%	7.7 ± 4.1%	7.8 ± 3.1%	8.1 ± 3.2%	12.3 ± 6.3%
CD34 (PPA-total)	6.1 ± 2.1%	5.8 ± 1.9%	7.1 ± 2.2%	6.1 ± 2.1%	5.2 ± 2.5%	4.4 ± 1.2%
CD34 (PPA-glomerulus)	14.0 ± 3.6%	14.0 ± 5.1%	14.6 ± 5.2%	13.0 ± 4.8%	8.9 ± 4.9%	9.7 ± 2.7%
CD34 (PPA- peritubular capillary)	4.7 ± 1.9%	4.2 ± 2.2%	4.8 ± 1.8%	4.5 ± 2.1%	4.0 ± 2.1%	3.9 ± 1.3%

CKD: chronic kidney disease; BMI: body mass index; SBP: Systolic blood pressure; DBP: Diastolic blood pressure; eGFR: estimated glomerular filtration rate; PPA: percentage of positive area; LK: left kidney; RRI: renal resistive index.

### RMD across different CKD stages

An independent samples t test revealed a slight increase in RMD (CD34 PPA-total and CD34 PPA-peritubular capillary) from CKD stages 1 to 2, while there was no change in CD34 PPA-glomerulus (Table 3). Subsequently, RMD (CD34 PPA-total, CD34 PPA-peritubular capillary, and CD34 PPA-glomerulus) gradually declined with disease progression from CKD stages 2 to 5 (Table 2, Figure 4).

**Table 3.** CD34 PPA comparison of CKD1 and CKD2 (left kidney).

CKD1 vs CKD2	*t* value	*p* Value
CD34 (PPA-total)	−2.22	0.031
CD34 (PPA-glomerulus)	−0.9	0.372
CD34 (PPA- peritubular capillary)	−2.673	0.010

PPA: percentage of positive area; CKD: chronic kidney disease.

### Correlation between the eGFR and fibrosis and between the eGFR and the RMD

**Figure 5.**
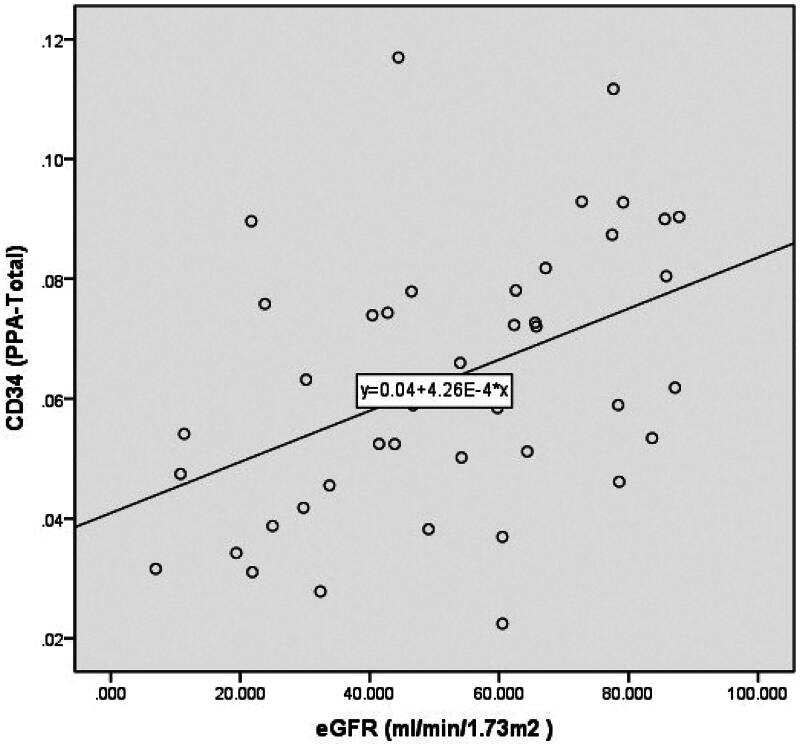
Scatter diagram showing the eGFR and CD34-stained areas (PPA-total) with disease progression from CKD stages 2 to 5. CKD: chronic kidney disease; eGFR: estimated glomerular filtration rate; PPA: percentage of the positive area.

**Figure 6.**
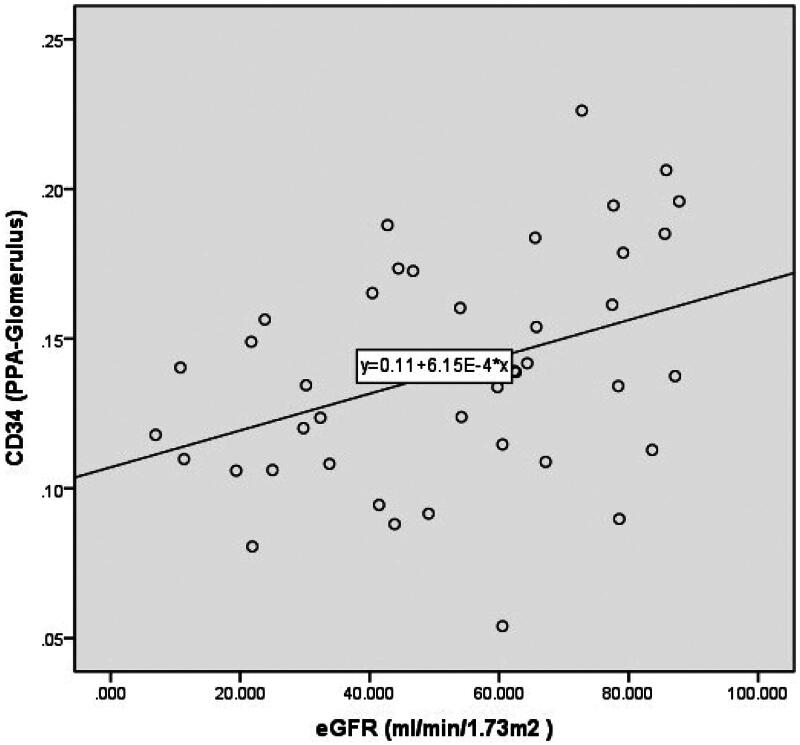
Scatter diagram showing the eGFR and CD34-stained areas (PPA-glomerulus) across CKD stages 2 to 5. CKD: chronic kidney disease; eGFR: estimated glomerular filtration rate; PPA: percentage of the positive area.

**Figure 7.**
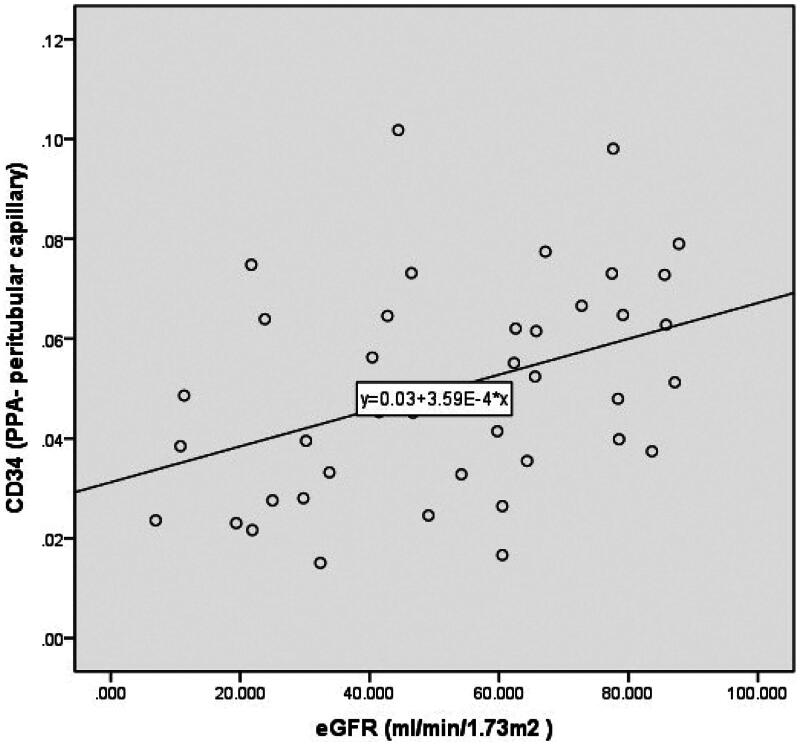
Scatter diagram showing the eGFR and CD34-stained areas (PPA-peritubular capillary) across CKD stages 2 to 5. CKD: chronic kidney disease; eGFR: estimated glomerular filtration rate; PPA: percentage of the positive area.

The eGFR demonstrated a negative correlation with the Masson PPA (total, glomerulus, or peritubular capillary) as disease progressed from CKD stages 1 to 5 (Table 4). Conversely, a positive correlation was observed between the eGFR and CD34 PPA (total, glomerulus, and peritubular capillary) from CKD stages 2 to 5 (Figures 5–7). However, no correlation was found between the eGFR and CD34 PPA (total, glomerulus, and peritubular capillary) from CKD stages 1 to 5.

**Table 4.** Correlation between RRI and other factors and between eGFR and other factors.

Characteristic	Spearman rho (*p* Value)			
Basic data	RI(CKD1-5)	eGFR(CKD1-5)	RI(CKD2-5)	eGFR(CKD2-5)
Age (years)	0.432(0.000)	−0.102(0.393)	0.447(0.003)	0.152(0.338)
BMI (kg/cm2)	0.081(0.497)	−0.148(0.210)	0.313(0.043)	0.174(0.269)
SBP(mmHg)	0.033(0.782)	−0.237(0.043)	0.061(0.699)	−0.073(0.648)
DBP(mmHg)	−0.104(0.379)	−0.259(0.027)	−0.179(0.256)	−0.181(0.252)
Left kidney lenth(cm)	−0.132(0.265)	0.222(0.059)	−0.057(0.718)	0.256(0.102)
eGFR (ml/min/1.73m^2^)	−0.044(0.710)	/	0.097(0.541)	/
Inter lobular artery RRI of LK	/	−0.044(0.710)	/	0.097(0.541)
**Pathological data(LK)**				
Masson (PPA-total)	−0.137(0.247)	−0.316(0.006)	−0.088(0.581)	−0.280(0.073)
Masson (PPA-glomerulus)	−0.129 (0.276)	−0.249(0.034)	−0.122(0.440)	−0.199(0.206)
Masson (PPA- peritubular capillary)	−0.201(0.088)	−0.328(0.005)	−0.163 (0.302)	−0.299(0.054)
CD34 (PPA-total)	−0.133(0.262)	0.077(0.517)	−0.232(0.139)	0.469(0.002)
CD34 (PPA-glomerulus)	−0.179(0.129)	0.157(0.185)	−0.353(0.022)	0.382(0.013)
CD34 (PPA- peritubular capillary)	−0.096(0.418)	0.028(0.813)	−0.211(0.179)	0.426(0.005)

CKD: chronic kidney disease; BMI: body mass index; SBP: Systolic blood pressure; DBP: Diastolic blood pressure; eGFR: estimated glomerular filtration rate; PPA: percentage of positive area; LK: left kidney; RRI: renal resistive index.

### Correlation between RRI and other parameters[AQ]

**Figure 8.**
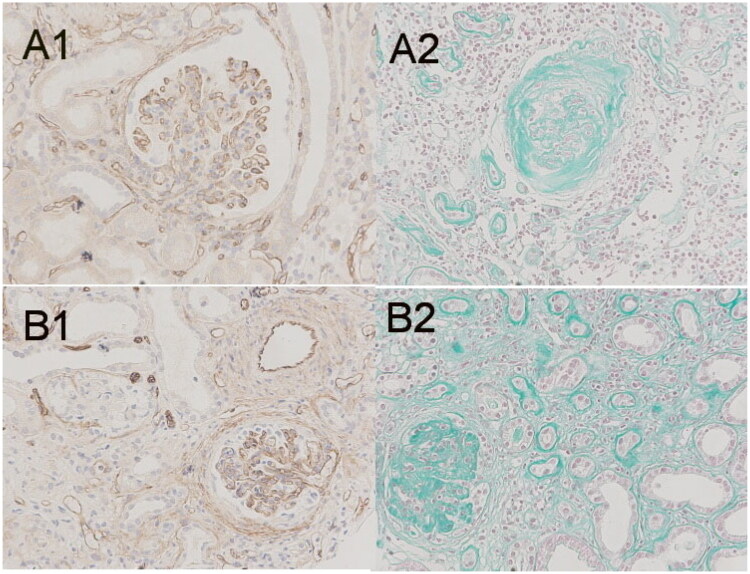
Optical microscopy showing the degree of fibrosis and microvessel density in CKD patients of different stages. A was a 62 year old patient with diabetes nephropathy. Patient A's eGFR was 53.98 ml/min/1.73m ^2^, the percentage of fibrosis area was 13.2%(A2), the CD34 PPA-total was 6.6%, the CD34 PPA-glomerulus was 11.4%, the CD34 PPA-peritubular capillary was 4.0%(A1), and the RRI was 0.61; B was a 25 year old patient with IgA nephropathy. Patient B's eGFR was 10.77 ml/min/1.73m ^2^, the percentage of fibrosis area was 37.2%(B2), the CD34 PPA-total was 4.7%, the CD34 PPA-glomerulus was 9.0%, the CD34 PPA-peritubular capillary was 2.4%(B1), and the RRI was 0.49. CD34 PPA-total:the percentage of the positive area of total area; RRI: resistive index;eGFR:the estimated glomerular filtration rate

With disease progression from CKD stages 1 to 5, the RRI exhibited a positive correlation with age but not with the CD34 PPA (total, glomerulus, or peritubular capillary) or the Masson PPA (total, glomerulus, or peritubular capillary) (Table 4, Figure 8).

**Figure 9.**
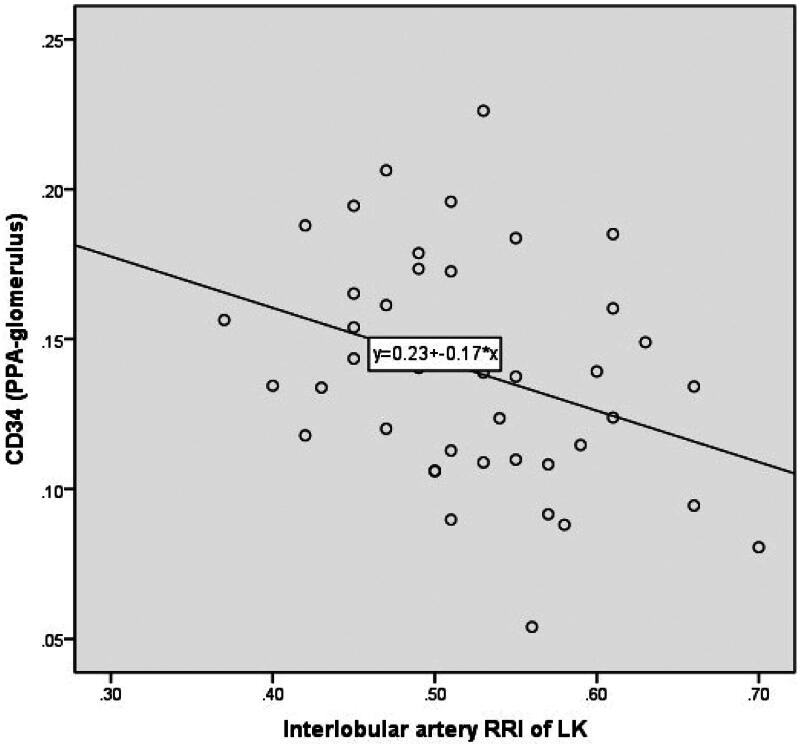
Scatter diagram showing CD34-stained areas (PPA-glomerulus) and the RRI of the interlobular artery across CKD stages 2 to 5. CKD: chronic kidney disease; PPA: percentage of the positive area; RRI: renal resistive index.

The RRI was negatively correlated with the CD34 PPA-glomerulus (Figure 9), positively correlated with age, and was not correlated with the CD34 PPA-total, CD34 PPA-peritubular capillary, or Masson PPA (total, glomerulus, or peritubular capillary) with disease progression from CKD stages 2 to 5.

### Correlation between the fibrosis degree and RMD of different CKD stages

There was no correlation between the degree of fibrosis and RMD (Table 5).

**Table 5.** Correlation between the fibrosis degree and microvessel density of different CKD stages.

	Spearman rho (*p* Value) of CKD 1–5	Spearman rho (*p* Value) of CKD 2–5
CD34 (PPA-total) and Masson (PPA-total)	0.007(0.952)	−0.031(0.847)
CD34 (PPA-glomerulus) and Masson (PPA-glomerulus)	0.100(0.398)	0.149(0.346)
CD34 (PPA- peritubular capillary) and Masson (PPA- peritubular capillary)	0.063(0.599)	0.059(0.709)

PPA: percentage of positive area.

## Discussion

The kidney is a highly vascularized organ with diverse endothelial cell (EC) functions throughout its different regions. Glomerular ECs serve as a filtration barrier and support podocyte structure, while peritubular capillary ECs transport reabsorbed components. Large and small vessel ECs maintain the renal vasculature [19]. Endothelial injury caused by toxins, immune cell activity, thrombosis, and inflammation can lead to acute kidney diseases or CKD. CD34 is an important marker for vascular ECs, and previous research has shown a higher number of interstitial capillaries stained with CD34 compared to CD31 in healthy kidneys [20]. Therefore, we utilized CD34 as an EC marker in our study.

Although a previous study reported a negative correlation between the resistive index (RRI) and renal microvascular density (RMD) in the peritubular capillary [17], their investigation did not include patients across different CKD stages and had a small sample size of 30 patients. In our study, we thoroughly analyzed RMD across different CKD stages and found that RMD (CD34 PPA-total and CD34 PPA-peritubular capillary) significantly increased (*p* < 0.05) during disease progression from CKD stages 1 to 2, followed by a gradual decline across CKD stages 2 to 5. Furthermore, we observed that eGFR was positively correlated with RMD (CD34 PPA-total, CD34 PPA-peritubular capillary, and CD34 PPA-glomerulus) during disease progression from CKD stages 2 to 5 (*r* = 0.469, 0.382, 0.426, respectively). However, no correlation was found between eGFR and these parameters from CKD stages 1 to 5. Based on these findings, we recommend initiating investigations into the correlation between RMD and contributors to CKD starting from CKD stage 2.

In terms of the RRI, we observed no correlation with CD34 PPA-total, CD34 PPA-peritubular capillary, CD34 PPA-glomerulus, or eGFR during disease progression from CKD stages 1 to 5 (Table 4). However, the RRI displayed a negative correlation with the CD34 PPA glomerulus (r=-0.353) from stages 2 to 5. Our results indicate that progressive CKD (worsening from stages 2 to 5) is accompanied by increased and severe endothelial cell injury, resulting in reduced glomerular ECs and blood flow. The elevated RRI due to vascular endothelial injury can be attributed to factors such as capillary or small-vessel thrombosis with fibrin deposition and swollen ECs that obstruct blood flow in glomerular capillaries or small arteries. Renal arteriosclerosis also contributes to the increase in RRI. Some studies have reported a correlation between the RRI and renal arteriosclerosis [16,21–23]. A study found similar RRI values in patients with moderate or normal intimal thickening (intima/media ratio <1) (0.62 vs. 0.60, *p* = 0.71). In contrast, patients with severe arteriosclerosis (intima/media ratio ≥1) had a significantly higher RRI than patients with moderate or no arteriosclerosis (0.73 vs. 0.61, *p* = 0.032) [16]. The aforementioned factors resulted in reduced kidney blood flow, and the decreased diastolic blood flow velocity of the renal artery led to a high RRI, which serves as the mechanism underlying the negative correlation between the RRI and CD34 PPA-glomerulus from CKD stages 2 to 5 observed in the present study. However, in our study, the RRI was not correlated with the CD34 PPA-total or CD34 PPA-peritubular capillary from CKD stages 2 to 5, a result that is attributable to the diverse composition of the study samples (the RRI values were ≤0.7 in all patients; patients with CKD4-5 were fewer; in the composition of patients with CKD, the glomeruli are mainly damaged or the glomeruli are invaded first).

Fibrosis is an important histopathological manifestation of CKD progression. Many studies have reported a strong correlation between kidney interstitial fibrosis and the RRI [16,17,22,24]. However, studies have given rise to conflicting conclusions regarding the correlation between the RRI and glomerulosclerosis. Some authors have refuted a correlation between these variables [16,23], whereas others have opposing views [25,26]. In the present study, no correlation was found between the RRI and Masson PPA (total, glomerulus, or peritubular capillary) from CKD stages 1 to 5. The differences between our results/conclusions and those of the aforementioned studies are attributable to the diverse composition of the study samples. In addition, the eGFR was negatively correlated with the Masson PPA across CKD stages 1 to 5 in the study.

There was no correlation between the degree of fibrosis and RMD in our study. However, as the disease progressed, the former showed an upward trend, while the latter showed trend of first increasing and then decreasing. The reason may be that there were relatively more CKD stage 1–3 patients among the study subjects.

We observed a correlation between RRI and age in our study, which concurs with the findings of many previous studies [27–29]. Aging is associated with increased vascular sclerosis, with a consequent increase in the RRI.

Several factors affect RRI, including systemic pulse pressure [2], aortic stiffness [3], renal artery stenosis, and renal interstitial and venous pressure [30–31]. In our study, we excluded patients with renal artery stenosis, abdominal aortic stenosis, hydronephrosis, and renal vein thrombosis among other such conditions at the experimental design stage to minimize the effect of factors that affect the RRI.

The limitations of this study are as follows: (i) this small-scale study included few patients with CKD stages 4 and 5 (*n* = 9) because patients in CKD4 and CKD5 stages rarely undergo kidney puncture; (ii) the RRI in this study was ≤0.7; (iii) we did not rule out thoracic aortic stenosis and abnormal cardiac hemodynamics in the patients included in our study. Therefore, large-scale studies that include patients with CKD stages 4 and 5 and RRI >0.7 are warranted in the future. However, this is the first study regarding the correlation between glomerular RMD and the RRI, providing useful guidelines to encourage further research in this domain. Although we did not observe a correlation between the RRI and RMD in our study, it is reasonable to assume that a low RRI (≤0.7) mainly affects glomerular blood supply in most CKD patients, an elevated RRI gradually affects the peritubular capillary and that the RRI may be correlated with the CD34 PPA-peritubular capillary and CD34 PPA-total. Therefore, the RRI may be a useful early predictor of glomerular vascular endothelial disease. In addition, for the first time, we found that the RMD (CD34 PPA-total and CD34 PPA-peritubular capillary) increased (*p* < 0.05) with disease progression from CKD stages 1 to 2, and the RMD gradually declined across CKD stages 2 to 5. Based on some studies, causes of increased blood flow in the early stages of CKD have been suggested; that is, there is an early proliferative response of the peritubular capillary endothelium [32–33]. However, further research is required to conclusively corroborate our findings.

In summary, the conclusion of our research was that there was no correlation between the RRI and RMD or between the RRI and fibrosis in CKD patients across stages 1 to 5 (RRI ≤ 0.7).
